# Procyanidin B2 and an autochthonous apple pulp extract modulate oxidative stress and PPARγ expression on an in vitro model of lipid steatosis in HepG2 cells

**DOI:** 10.1007/s13105-026-01151-9

**Published:** 2026-01-23

**Authors:** Adrián Millán-Laleona, Marta Lopez-Yus, Silvia Lorente-Cebrián, Jose M. Arbones-Mainar, Carlota Gómez-Rincón, Víctor López

**Affiliations:** 1 Department of Pharmacy, Faculty of Health Sciences, Universidad San Jorge, Zaragoza, 50830 Spain; 2 Instituto Agroalimentario de Aragón-IA2, CITA-Universidad de Zaragoza, Zaragoza, 50013 Spain; 3 Instituto de Investigación Sanitaria (IIS) Aragon, Zaragoza, 50009 Spain; 4 Adipocyte and Fat Biology Laboratory (AdipoFat), Translational Research Unit, University Hospital Miguel Servet, Zaragoza, 50009 Spain; 5 Instituto Aragones de Ciencias de la Salud (IACS), Zaragoza, Spain; 6 Centro de Investigación Biomédica en Red Fisiopatología Obesidad y Nutrición (CIBERObn), Madrid, 28029 Spain; 7 Departamento de Farmacología, Fisiología y Medicina Legal y Forense, Universidad de Zaragoza, Instituto Agroalimentario de Aragón (IA2), Universidad de Zaragoza-CITA), Zaragoza, Spain

**Keywords:** MASLD, Lipid metabolism, Phenolic compounds, Procyanidin B2, Apple, HepG2

## Abstract

Metabolic dysfunction-associated steatotic liver disease (MASLD) is characterized by hepatic steatosis alongside metabolic comorbidities like type 2 diabetes mellitus (T2DM), dyslipidaemia, overweight, or obesity. Liver damage could be prevented promoting consumption of fruits and vegetables, the main source of phenolic compounds. These molecules have been previously related with antioxidant and anti-obesity properties. Therefore, the objective of this study is to determine the antioxidant capacity and potential preventive activity of an autochthonous apple pulp extract, Amarilla de Octubre, and its main phenolic compound, procyanidin B2 (PB2), in an in vitro model of liver steatosis. Antioxidant activity was assessed by xanthine/xanthine oxidase (X/XO) system and nitric oxide scavenging production. Monoamine oxidase A (MAO-A) inhibition was also determined due to its implication in detoxification processes in liver. Human hepatocytes (HepG2 cell line) were selected to test their potential cytotoxicity, oxidative stress situations (ROS production) and the potential effect in lipid metabolism by overloading cells with oleic acid. Fat accumulation and gene expression related to lipid metabolism (PPARγ, CD36 and FAS) were also analysed by Oil Red O and qPCR respectively. PB2 showed promising results as an antioxidant compound in scavenging and enzymatic inhibition-related experiments. However, Amarilla de Octubre not only reduce fat accumulation, but also modulates the expression of PPARγ, CD36, and FAS and demonstrates slightly lower efficacy than PB2 in scavenging and enzymatic inhibition-related experiments. These results highlight the potential of apples to mitigate pathogenic conditions associated with metabolic disorders such as obesity, emphasizing the role of dietary polyphenols in this protective effect.

**Supplementary Information:**

The online version contains supplementary material available at 10.1007/s13105-026-01151-9.

## Key points

The polyphenol procyanidin B2 exhibits strong antioxidant and MAO-A inhibitory potential, showing promising results for influencing the development of lipid accumulation in hepatocytes.

Procyanidin B2 and Amarilla de Octubre apple pulp extract protect human HepG2 hepatocytes from hydrogen peroxide-induced stress by attenuating intracellular ROS production.

The pulp extract from the Amarilla de Octubre apple reduces fat accumulation in oleic-acid overloaded HepG2 cells. Both tested samples, procyanidin B2 and the extract, modulate the expression of PPARγ, CD36, and FAS reducing their mRNA expression.

**Supplementary Information:**

The online version contains supplementary material available at 10.1007/s13105-026-01151-9.

## Introduction

The term metabolic dysfunction-associated steatotic liver disease (MASLD) was recently introduced to describe the former fatty liver disease linked to systemic metabolic dysregulation. This evolution in terminology from classic non-alcoholic fatty liver disease (NAFLD) to metabolic dysfunction-associated fatty liver disease (MAFLD) and ultimately MASLD, includes a set of criteria designed to facilitate diagnosis for medical community and to be non-stigmatizing [1, 2].

With the rising global incidence of obesity, the clinical and economic load of MASLD is expected to increase substantially [3]. Obesity has also been linked to type 2 diabetes mellitus (T2DM), a significant risk factor for MASLD, which appears to accelerate the progression of liver disease [4]. To prevent serious associated complications such as cirrhosis, or hepatocellular carcinoma it is important to arrest disease progression [5].

Although MASLD is generated by the fat metabolism imbalance because of the abnormal accumulation of lipids in liver tissue, no specific drugs have been determined for the treatment of this affection. It is now recognized as the result of multiple contributing factors, including insulin resistance, adipose tissue-derived hormones, nutritional influences, genetic and epigenetic factors, as well as microbiota disruption [6, 7]. Alterations in the expression of certain detox enzymes such as MAO [8] or xanthine oxidase (XO) [9] may be considered to evaluate the severity of liver steatosis. Thus, increased levels of reactive oxygen species (ROS) and lipid peroxidation contribute to cellular stress and promote a pro-inflammatory state through mitochondrial damage [10]. Metabolic regulators of hepatic lipid metabolism such as peroxisome proliferator-activated receptors (PPARs) [11], some free acid lipids transporters [12] or fatty acid synthase (FAS) [13] play important roles regulating energy balance and fibrosis development in liver. Emerging approaches targeting the gut microbiota or the enterohepatic axis are gaining attention. However, dietary modifications remain the most effective non-drug based strategy against MASLD [14].

Reducing lipid concentration intake and exercise intervention are the main objectives to promote the regression of steatosis [15]. Considering these approaches, dietary polyphenols represent a significant group of compounds with potential therapeutic effects against liver steatosis and steatohepatitis [16]. Fruits, vegetables, or even coffee are sources of those molecules with interesting benefits for human health [17]. Moreover, the Mediterranean diet is well characterised by promoting the consumption of polyphenol rich foods and represent one of the most effective applications to restrain fatty liver disease conditions [18]; particularly, a flavonoid-rich diet has been linked to the reduction of certain metabolic and cardiovascular risk [19, 20].

Apple fruits have been recognized as a valuable source of flavonoids. Those phytochemicals are widely present in their pulp and skin, differing between apple cultivars. Factors such as variety, climatology or stage of maturity influence directly in their phytochemical composition [21]. Previous experiments in our group found procyanidin B2 (PB2) as the major polyphenol in apple pulp samples of some autochthonous (local/regional) apple accessions [22] with interesting in vitro bioactive properties [23]. PB2 is the major isomers of procyanidins, consisting of two molecules of (-)-epicatechin and it is considered an edible pigment abundantly available in fruits such as apples, pears and peaches [24]. Health benefits have been associated to this natural flavonoid, particularly metabolic disorders [25]. PB2 has been linked to anti-oxidant, anti-inflammation, and lipid metabolism regulation properties [24]. Thus, previous studies have shown protective effects of this particular polyphenol against liver fibrosis mediated by the Hedgehog pathway, responsible for tissue remodelling and maintenance, an important mediator in various liver injuries [26].

In the present study, we evaluated the impact of the apple pulp extract from the Spanish accession known as Amarilla de Octubre and its main polyphenol, procyanidin B2, as potential in vitro modulators for MASLD pathogenesis. The experiments were focused on their scavenging properties, MAO-A and xanthine oxidase inhibitory activity, effects on intracellular ROS production in human hepatocytes, fat accumulation and modulation of RNA expression of key regulators involved in adipose tissue metabolism, including PPARγ, CD36, and FAS.

## Materials and methods

### Samples

Procyanidin B2 was acquired from Extrasynthese (Lyon, France). Apples were collected from mountainous areas of the Pyrenees (Spain) and recovered in collections located in Garcipollera (Huesca, Aragon). Selected apple fruits were collected between August and October at the ripening period of the cultivar. The pulp tissue (500 g) was frozen immediately and lyophilized. The extraction of bioactive compounds was achieved by using methanol in a 33:1 (v/w) mixture of solvent: lyophilized ground tissue and ultrasonication for 20 min at room temperature. Extract was stored at − 80 °C after elimination of solvent in a rotatory evaporator. Procyanidin B2 was tested as the predominant phenolic compound because it was previously reported in the Amarilla de Octubre pulp extract by using analytical techniques like HPLC MS/MS; the main phenolic compounds were the following expressed in mg/g extract: procyanidin B2 (70.23), epicatechin (18.03), 3-caffeoylquinic acid (15.65), catechin (3.49) and other minor compounds. We attach the reference paper: [22].

### Radical scavenging activity and enzyme Inhibition of xanthine/xanthine oxidase (X/XO) system

The effects of the samples on the X/XO system were measured by the NBT (nitro tetrazolium blue chloride) (Sigma-Aldrich, Barcelona, Spain) radical superoxide (O_2_•) complex formation. The assay was developed using xanthine oxidase from bovine milk (Sigma-Aldrich, Barcelona, Spain). 16 mM Na_2_CO_3_ (Fisher Scientific, Barcelona, Spain), 22.8 µM NBT and 90 µM xanthine (Sigma-Aldrich, Barcelona, Spain) were dissolved in phosphate buffer NaH_2_PO_4_ (Fisher Scientific, Barcelona, Spain) 18 mM pH 7 to reach a volume of 240 µL on each well of a 96 microplate. Then, 30 µL of each sample, previously dissolved in the phosphate buffer, and 30 µL of xanthine oxidase (168 U L^− 1^) were added to start the reaction. Assay mixtures were allowed to react during 2 min at 37 °C. After this incubation, plates were measured at 560 nm in a Synergy H1 reader (BioTek^®^ Instruments, Inc., Winooski, VT, USA) [27]. Blank samples were also considered, and percentages of inhibition were calculated according to Eq. (1):

$$\:Inhibition\:\left(\%\right)=\left[\frac{\left(Abs\:control-Abs\:sample\right)}{Abs\:control}\right]*100$$

PB2 and Amarilla de Octubre apple Pulp extract were also assessed to determine their inhibitory potential of XO. As described previously, 16 mM Na_2_CO_3_ (Fisher Scientific, Barcelona, Spain), and 90 µM xanthine (Sigma-Aldrich, Barcelona, Spain), without NBT, were dissolved in phosphate buffer NaH_2_PO_4_ (Fisher Scientific, Barcelona, Spain) 18 mM pH 7. After adding the enzyme (also 168 U L^− 1^) to the mixtures and the reaction time was completed at 37 °C, plates were measured at 295 nm.

### Nitric oxide scavenging assay

Different concentrations of the samples were dissolved in phosphate buffer 0,1 M pH 7.4 and mixed with the substrate sodium nitroprusside dihydrate (Sigma Aldrich). They were incubated 60 min at room temperature under a source of light. Griess reagent (Sigma Aldrich) was added to the mixture after the incubation. The absorbance was measured at 560 nm after 10 min of dark incubation. Quercetin (Fluorochem, Barcelona, Spain) was performed as positive control. The procedure is based on the assay by Sreejayan & Rao [28].

### MAO-A inhibition

Samples were analysed for MAO-A inhibition as previously reported by Olsen and collab (Olsen et al., 2008). Each well contained designated range of concentrations for apple pulp extract, PB2 or buffer (control), chromogenic solution (0.8 mM vanillic acid, 417 mM 4-aminoantipyrine and horseradish peroxidase (4 U mL − 1) in potassium phosphate buffer pH = 8), tyramine (0.2 M) and MAO-A (8 U mL − 1) [29]. Clorgyline was employed as the reference drug [30]. The absorbance was read at 490 nm every 5 min for 30 min in a Synergy H1 reader.

### Cell culture and cell viability

HepG2 (liver human cancer cell line) were purchased from ATCC. They were cultured in DMEM medium (Sigma Aldrich), 10% fetal bovine serum (FBS) (Sigma Aldrich), 1% Hepes (Sigma Aldrich) and 0.5% penicillin-streptomycin (Sigma Aldrich) in the presence of 5% CO_2_ at 37 °C. Cytotoxicity was evaluated using the Janus Green B method. HepG2 cells were seeded in 96-well plates at 3 × 10^4^ cel/ml. After 24 h, cells were treated with various concentrations of the apple extract or polyphenols for 24 h (from 62.5 to 250 µg/ml or from 25 to 100µM respectively). Janus Green B (JG) undergoes reductive splitting upon the action of electron transport chain (ETC) oxidoreductases of the actively respiring mitochondria to form diethyl safranin. Cell cultures were fixed with 10% formaldehyde at room temperature for, at least, 60 min. Then, plates were washed twice with PBS pH 7.4 and incubated with 0,3% JG solution in the dark for 5 min in agitation. Excess of colorant was eliminated washing 4 times with distilled H_2_O and let the plates dry. Then, HCl 0.5 M was added to dissolve the diethyl safranin formation. Plates were in agitation for 10 min and finally, absorbance was evaluated by measuring absorbance at 595 nm (Synergy HT, Biotek).

### Oleic acid-induced steatosis in human hepatocytes

HepG2 were cultured at 3 × 10^4^ cell/ml in 6 or 96-well plates as described before. After 24 h, cell culture medium was replaced by DMEM plus the apple extract or the polyphenol at the concentration of interest (from 62.5 to 250 µg/ml for the apple extract or from 25 to 100µM for the polyphenol) for another 24 h. Finally, for oleic-acid induced steatosis, DMEM medium with the extract or polyphenol was removed and, cells were exposed for another 72 h with DMEM medium supplemented with 0.5 M of oleic acid to induce lipid accumulation [31].

### ROS production

HepG2 cells were seeded at 3 × 10^4^ cell/ml in 96 well plates for 24 h. Then, medium was retired, and the cells were incubated for 20 min at 37 °C by using DCFH-DA as a fluorescent probe to determine intracellular reactive oxygen species (ROS). After the incubation, HepG2 were treated with different concentrations of PB2 and Amarilla de Octubre pulp extract plus hydrogen peroxide 500µM simultaneously. ROS generation was then measured at 480 nm of excitation and 510 nm of emission (Synergy H1) at 37 °C for 90 min [32]. 

The influence of the polyphenolic samples was also assessed in OA induced lipid accumulation in HepG2 cells. Hepatocytes were also seeded at 3 × 10^4^ cell/ml in 96 well plates for 24 h. Subsequently, the medium was retired and then added DMEM medium supplemented with 0.5 M of OA (Merck) for 72 h. Finally, this fatty acid rich medium was also retired and then added the DCFH-DA for incubation and subsequently, as described below, different concentrations of the samples and the hydrogen peroxide 500µM simultaneously. Finally, fluorescence measurement was performed equally at 480 nm of excitation and 510 nm of emission (Synergy H1) at 37 °C for 90 min.

### Fat accumulation: oil red O staining of cellular lipids

Oil Red O (ORO) staining was used to detect accumulation of fatty acids. HepG2 cells were fixed with 10% formaldehyde at room temperature for, at least, 60 min. After that, cells were washed with 1×PBS. Oil Red O solution was filtered, added to the cells and incubated for 15–20 min in agitation. Cells were washed with distilled H_2_O twice and isopropanol 100% was added to dissolve Oil red. Finally, the absorbance of ORO was measured at 500 nm with isopropanol 100% as blank (Synergy HT, Biotek).

### Gene expression analysis

Total RNA was isolated from HepG2 cells using TRIzol (Sigma) according to the manufacturer’s protocol. RNA samples were treated with RNase-Free DNase to remove genomic DNA and 1 µg of treated RNA was reverse-transcribed using PrimeScript Reverse Transcriptase (Takara Bio). cDNA product was diluted (1:10) and amplified by qPCR in a total volume of 15 µl per reaction with SYBR Select Master Mix (Applied Biosystems), 0.5 µl of gene-specific primers at 10µM (Table 1) for PPARγ, CD36 and FAS expression. cDNA amplification was conducted in StepOnePlus system (Applied Biosystems), using a protocol as follows: 95 °C for 10 min, 40 cycles of 15 s at 95 °C and 1 min at 60 °C, 15 s at 95 °C, 1 min at 60 °C, and 15 s at 95 °C. In each run, a no-template control was included as a negative control, and after each run, a melt curve was performed to confirm the specificity of designed primers. Relative gene expression was normalized to Actin (ACTB) expression using the 2^−ΔΔCT^ method. The sequences of primers used for qPCR analysis in this study are listed in supplementary materials Table S1.

### Statistical analysis

Each experiment was performed at least three times on at least in three different days and results were expressed as the mean ± standard error of the mean (SEM) of different assays. Results were expressed as the mean ± standard error of the mean (SEM) of different assays. GraphPad Prism v.6 software (GraphPad Software, San Diego, CA, USA) was used to perform data analyses. Bioactivity studies assays were statistically analysed by using ANOVA following Tukey´s post-test.

## Results

### Antioxidant potential: X/XO system and NO- scavenging activity

**Fig. 1:**
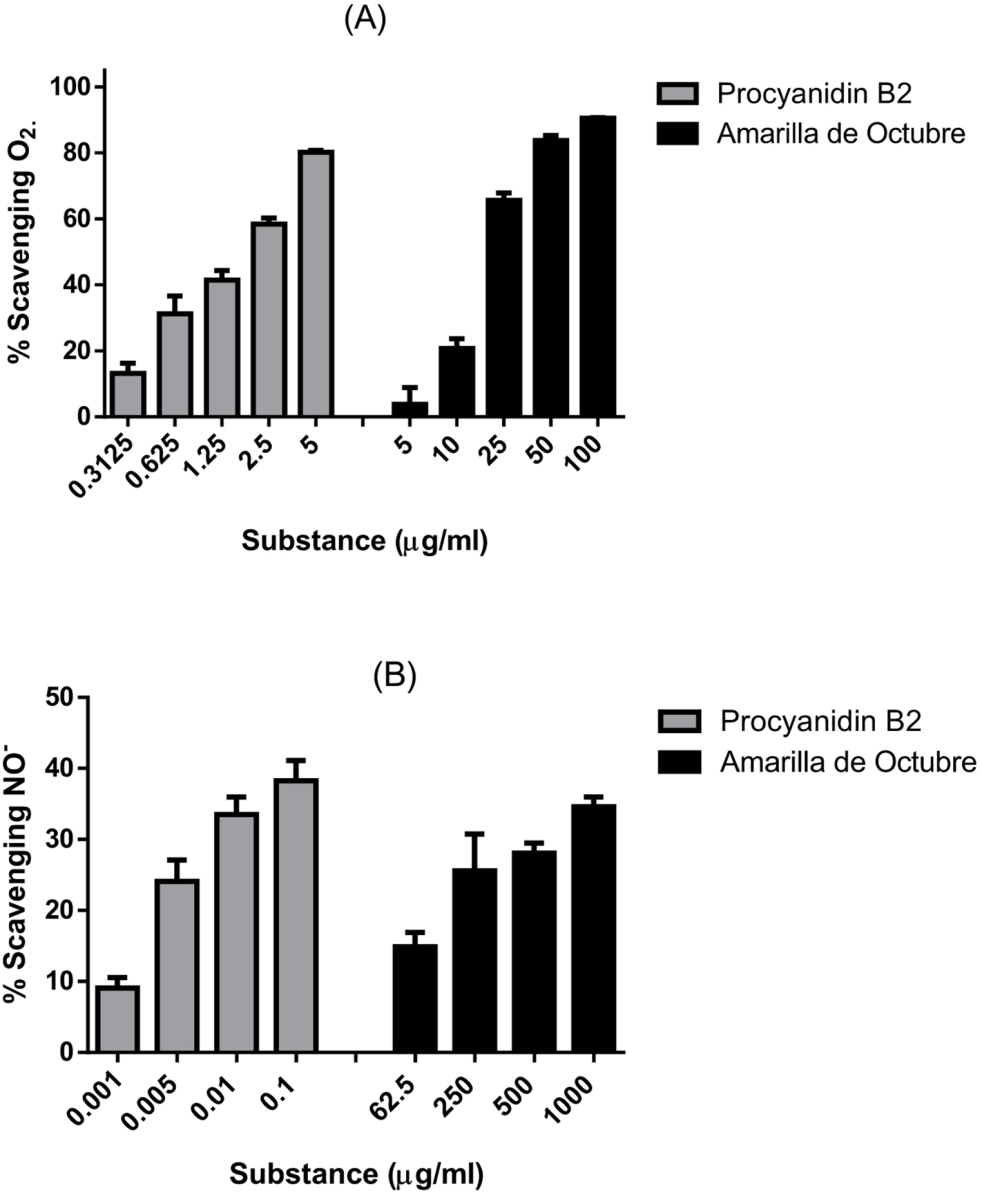
(**A**) Percentage of superoxide scavenging potential of PB2 and the Amarilla de Octubre pulp extract expressed in micrograms per millilitre. (**B**) NO − scavenging percentage expressed as the means of different concentrations in micrograms per millilitre of the extract. At least three independent replicates were performed per experiment

The antioxidant activity of PB2 and Amarilla de Octubre pulp extract were performed using different methods. In the X/XO scavenging assay, as shown in Fig. 1.A, the extract was not as effective as PB2 (Table 1). Thus, XO inhibition was also performed for both samples as a complementary assay. PB2 inhibited the enzyme with lower IC_50_ value than Amarilla de Octubre as showed in Table 1. The IC_50_ values were calculated by non-linear regression.

On the other hand, at tested concentrations, none of the samples reached IC_50_ values for the NO- scavenging assay, although PB2 achieved the maximum scavenging potential at 0.1 µg/mL (Fig. 1B).

**Table 1:** Results of the antioxidant activity based on X/XO system are expressed as average ± SEM of at least three independent experiments. IC_50_ was determined by using non-linear regression

IC_50_ values (µg/mL) in the X/XO system		
	Superoxide Scavenging	XO inhibition
Procyanidin B2	1.73 ± 0.14	199.80 ± 36.13
Amarilla de Octubre pulp extract	18.40 ± 1.29	1179.66 ± 31.60

### Monoamine oxidase A (MAO) inhibition

**Fig. 2:**
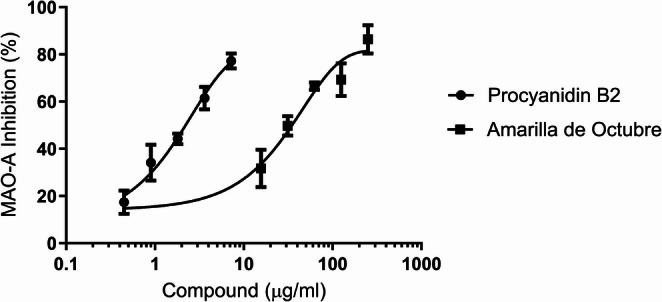
Monoamine oxidase A (MAO-A) inhibition potential of PB2 versus Amarilla de Octubre pulp extract. IC_50_ values were calculated by using non-linear regression. At least three independent replicates were performed

Figure 2 shows the inhibitory capacity of the isolated polyphenol, PB2 versus Amarilla de Octubre pulp extract on MAO-A inhibition. Both compounds reached IC_50_ values at tested concentrations (2.21 ± 0.16 µg/ml and 34.97 ± 2.48 µg/ml respectively). Clorgyline was also tested as positive control (data not shown).

### Cell viability

**Fig. 3:**
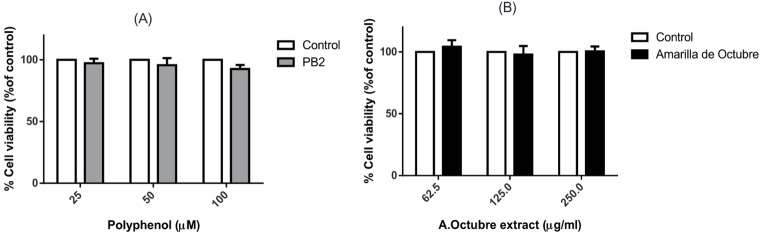
HepG2 expressed as percentages by using Janus green staining titration after incubating the cells for 24 h with PB2 (**A**) or apple pulp extract (**B**). Non-treated cells were considered as control group. No significant differences were found at none of the tested conditions in three independent replicates

As shown in Fig. 3, neither PB2 nor the Amarilla de Octubre pulp extract affected HepG2 at the tested concentrations, indicating a lack of cytotoxicity under these conditions. This result suggests that the treatments are not toxic for use in subsequent experiments aimed at evaluating their biological activities.

### ROS production in HepG2 cells

**Fig. 4:**
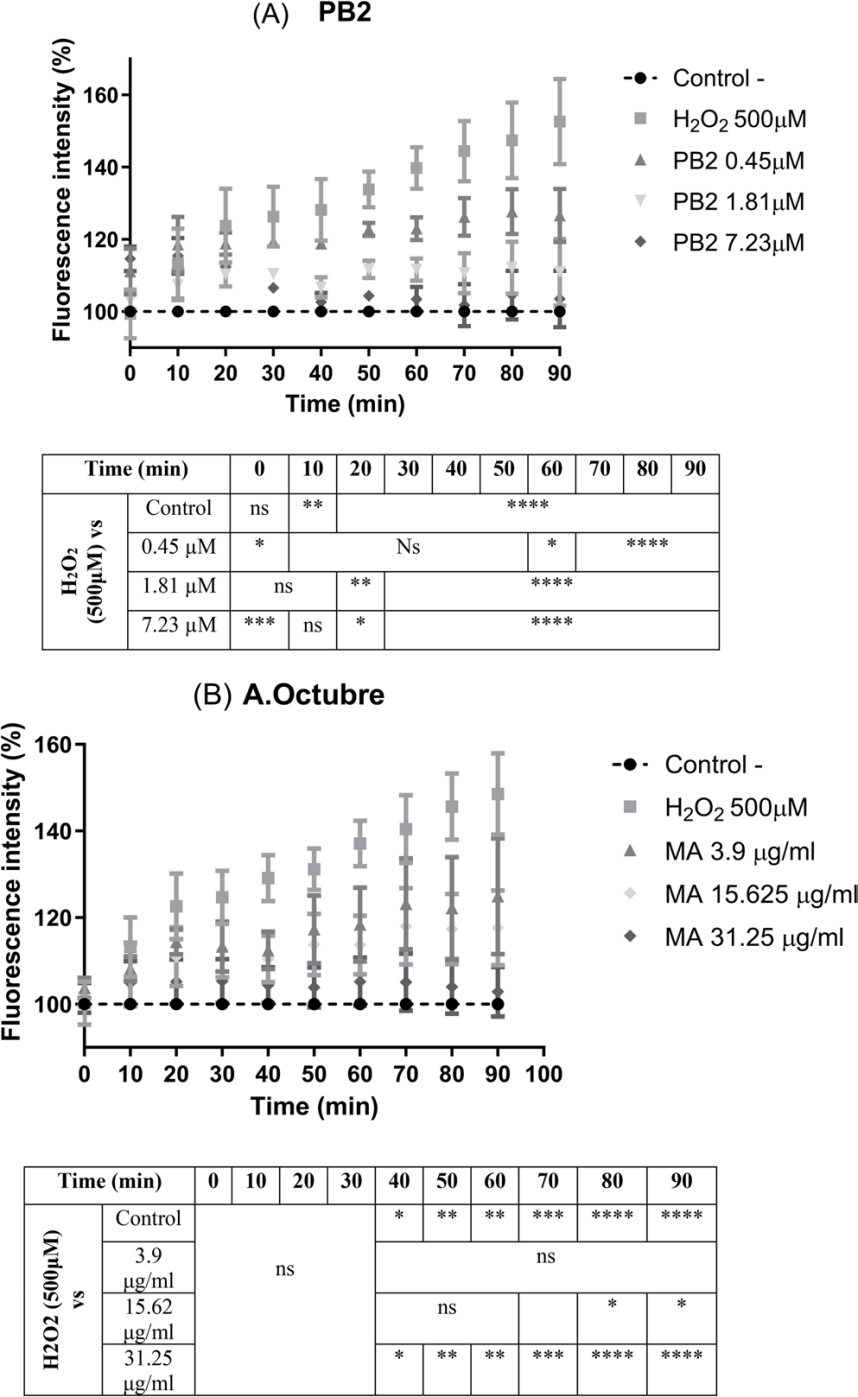
ROS production in HepG2 cells subjected to oxidative stress by hydrogen peroxide (500 µM) and coincubated with PB2 (from 0.45 to 7.23 µM) (**A**) or Amarilla de Octubre pulp extract (from 3.9 to 31.25 µg/ml) (**B**). Data were normalized and expressed as percentage over control cells without H_2_O_2_ and the assay was carried out for 90 min. ANOVA and Tukey’s post analysis was performed for the statistical analysis for at least three independent experiments; ns, * *p* < 0.1, **p < 0.01, ***p < 0.001, ****p < 0.0001 versus positive H_2_O_2_ treated cells

ROS production was measured for 90 min in HepG2 cells was determined in two cell different growing conditions. Figure 4 shows the ROS production in cells stressed by using 500 µM H_2_O_2_ and different concentrations of PB2 (A) or Amarilla de Octubre extract (B) as cotreatment.

**Fig. 5:**
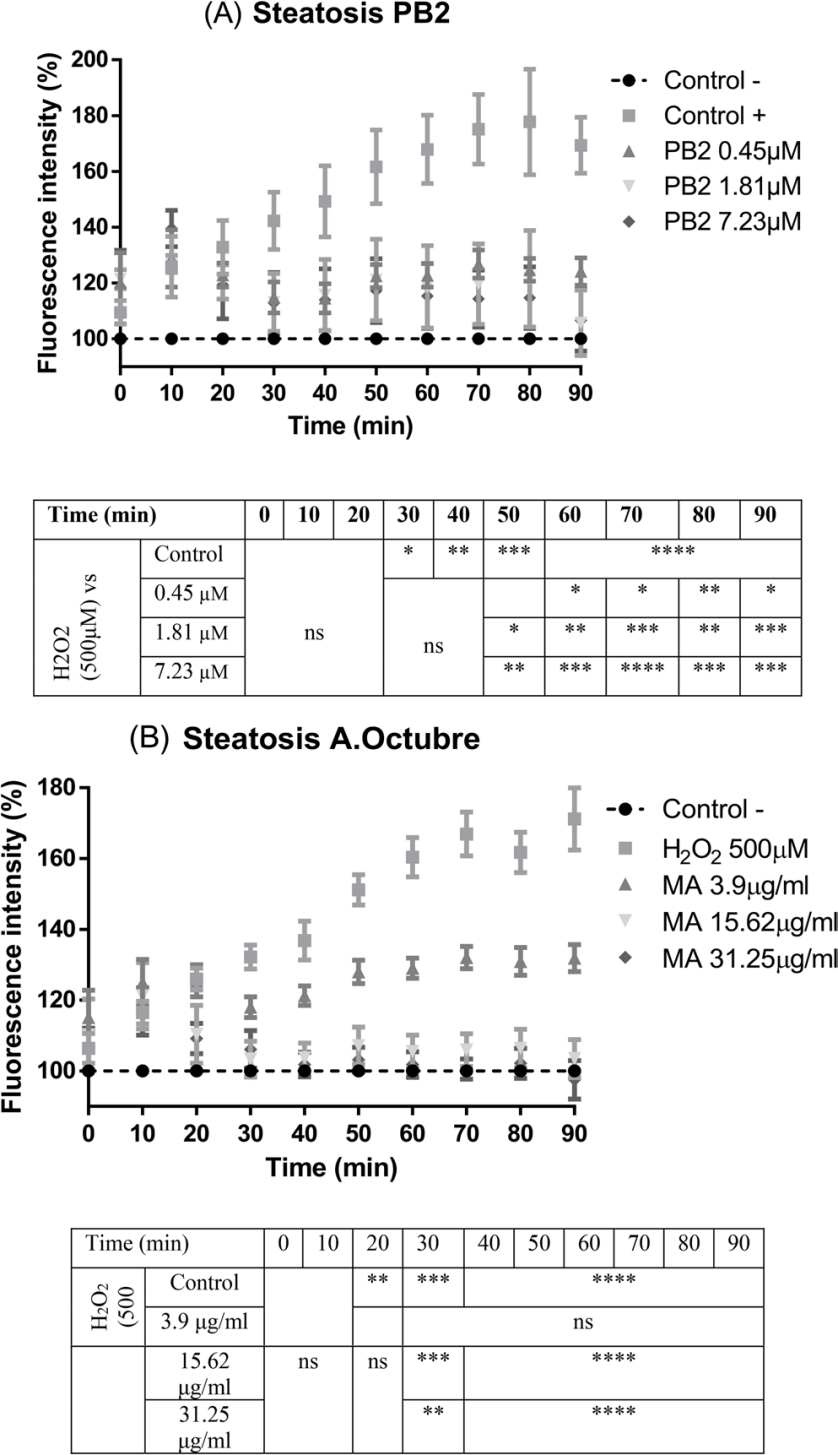
ROS intracellular production in OA preincubated HepG2 subjected to oxidative stress by hydrogen peroxide (500µM) and coincubated with PB2 (from 0.45 to 7.23 µM) (**A**) or the Amarilla de Octubre pulp extract (from 3.9 to 31.25 µg/ml) (**B**). Data were normalized and expressed as percentage over control cells without H_2_O_2_ and the assay was carried out for 90 min. ANOVA and Tukey’s post analysis was performed for the statistical analysis for at least three independent experiments; ns, * *p* < 0.1, **p < 0.01, ***p < 0.001, ****p < 0.0001 versus OA induced - H_2_O_2_ treated cells

In addition, Fig. 5 shows the results of OA induced HepG2 and then subjected to stress by 500µM H_2_O_2_ cotreated with the two compounds (PB2 and the apple pulp extract) as described in materials and methods section.

### Lipid accumulation: oil red O Titration

**Fig. 6:**
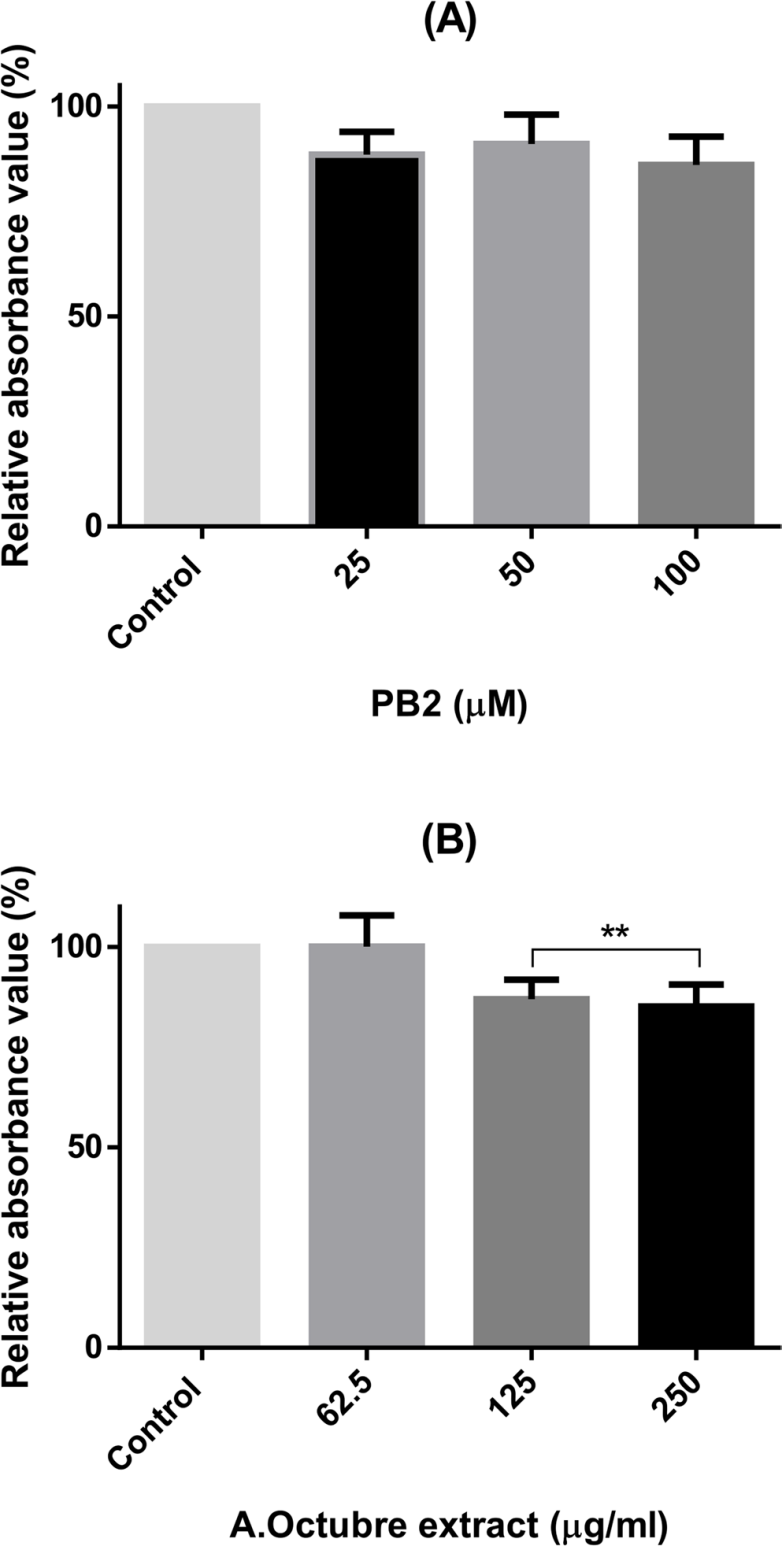
Oil red O quantification in HepG2 cells incubated with PB2 (**A**) and the apple pulp extract (**B**). ANOVA and Tukey’s post analysis was performed for the statistical analysis for at least three independent experiments; **p < 0.01 versus control group

Oil red O titration of HepG2 cells after the steatosis induction was used to determine the potential effect in lipid accumulation for PB2 and the apple pulp extract. No significant differences were found versus the control group for none of the tested concentrations of the isolated polyphenol (from 25 to 100µM) (Fig. 6A). However, statistical differences were found for 125 and 250 µg/ml of tested Amarilla de Octubre extract vs. the control group (Fig.6B).

### RNA expression levels: qPCR

**Fig. 7:**
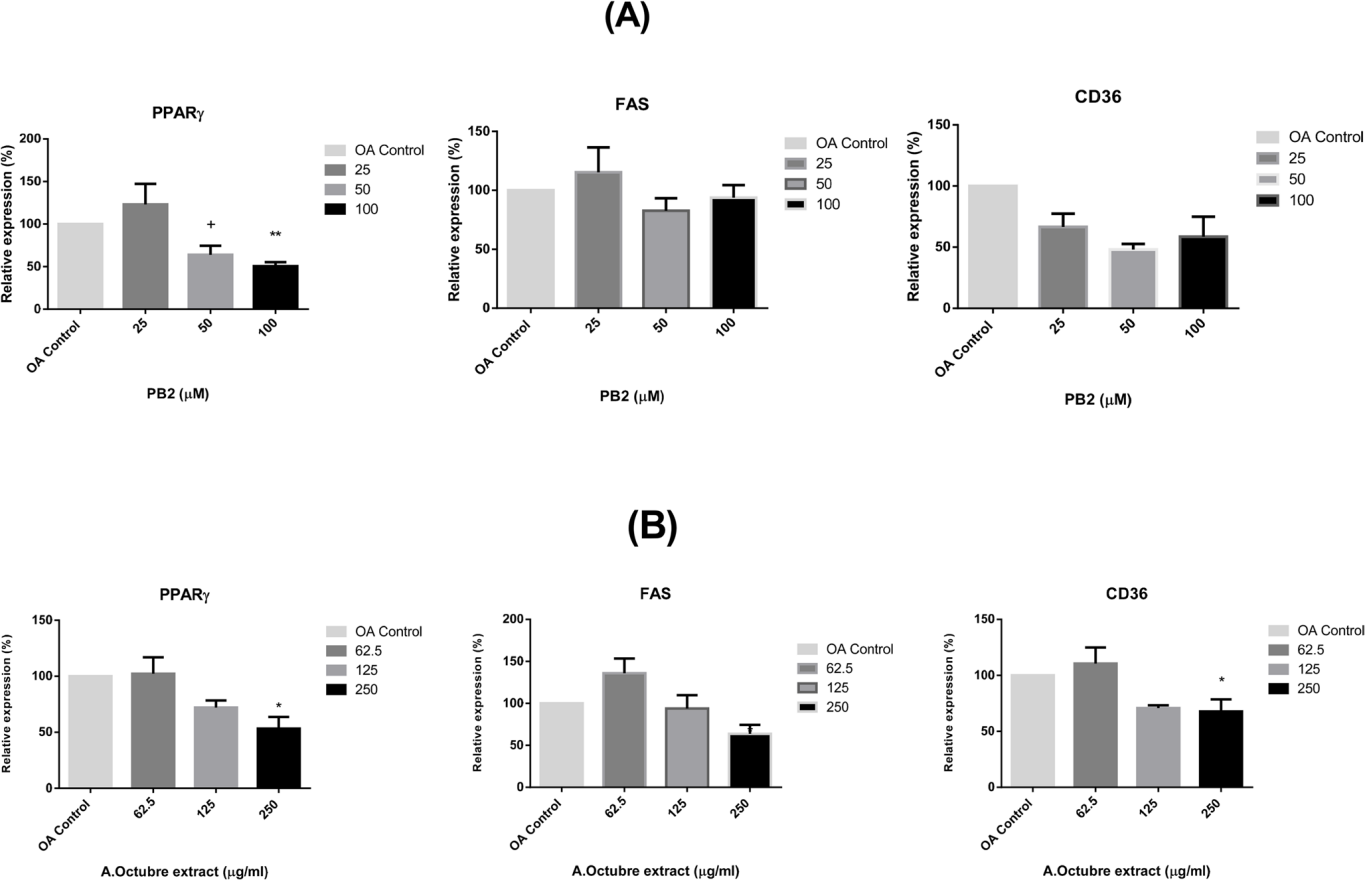
Relative RNA expression levels of three different fat metabolism related genes (PPARγ, FAS and CD36) after pretreatment with (**A**) PB2 at 25, 50 or 100µM (**B**) Apple pulp extract at 62.5,125 or 250 250 µg/ml and subsequently OA steatosis induction. Data were normalized over ACTB housekeeping gene, and it is expressed as percentage over OA control cells. ANOVA was performed for the statistical analysis for, at least, 3 independent replicates per condition; (**A**) ** *p* < 0.01 Significant differences between 100µM vs. 50µM, + *p* < 0.05 Significant differences between 50µM vs. 25µM; (**B**) * *p* < 0.05 Significant differences between 250 µg/ml vs. 62.5 µg/ml. Experiments were performed per triplicate in three different days

RNA levels of the transcription factor PPARγ, the receptor CD36, and the enzyme FAS were quantified using qPCR. As shown in Fig. 7(A), preincubation with procyanidin B2 at 100 µM significantly reduced the expression of PPARγ and caused a decrease in FAS and CD36 levels, although these reductions were not statistically significant.

On the other hand, Amarilla de Octubre pulp extract at its highest tested concentration (250 µg/ml) significantly reduced the expression of PPARγ and CD36 in HepG2 cells compared to the lowest tested concentration (62.5 µg/ml). Additionally, pretreatment with the apple extract also demonstrated a trend toward reduced FAS expression, as shown in Fig. 7B, highlighting its potential modulatory effect on fatty acid metabolism.

## Discussion

Preventing the development of steatosis in the liver is essential to avoid progressive damage to liver cells, as this damage can impair their ability to fully return to a healthy state. MASLD is often accompanied by metabolic alterations associated with conditions such as obesity, hyperlipidaemia, or diabetes. This pathological state causes cellular damage through increased production of intracellular ROS, leading to pro-oxidant conditions that promote oxidative stress and inflammation in hepatocytes [33].

Antioxidant mechanisms play a crucial role in MASLD. The presence of radical scavengers improves this pathogenesis by attenuation of cellular stress [34]. As a first approach, we evaluated, by X/XO method, the scavenging potential of PB2 and we compared it with Amarilla de Octubre pulp extract, obtaining as expected lower IC_50_ for the isolated polyphenol. Additionally, xanthine oxidase inhibition has been closely related with diminution in uric acid levels and attenuation of fat accumulation in liver cells [35]; thus, xanthine oxidase inhibitors could be considered as putative therapies against MASLD development, especially when hyperuricemia appears [36]. We observed the same trend comparing the IC_50_ calculated for the enzyme inhibition as well as the superoxide scavenging assay. In xanthine oxidase inhibition, also PB2 achieved lower IC_50_ than the apple pulp extract. These results underline the high antioxidant potential of the apple pulp extract and its main polyphenol, PB2.

Moreover, NO- radical scavenging activity was evaluated because of its linkage with inflammatory processes [37], as assessed in previous studies [22]. Nitrite scavenging showed again higher antioxidant potential for the isolated polyphenol. Another biochemical reaction generating oxidative stress and free radicals in the liver is monoamine oxidase. This enzyme, found in several human tissues, is related to detoxification properties and directly involved in peroxide production in the liver [38]; mitochondrial enzyme MAO-A modulates the generation of ROS by serotonin degradation and its overexpression in damaged liver tissues cause an important increase of hydrogen peroxide production [39]. All these facts and results obtained in the in vitro cell-free systems lead us to evidence if apple phenolic compounds and extracts could prevent ROS production in HepG2. As an antioxidant, procyanidin B2 can inhibit the generation of highly reactive species such as the hydroxyl radicals employed on this experiment to generate oxidative stress in cells [40, 41]. Regarding those results, apple extracts and PB2 were explored in HepG2 subjected to oxidative stress both in “standard” HepG2 and in oleic acid induced lipid accumulation. Significant differences versus de H_2_O_2_ group were also found for the apple pulp extract and the polyphenol as well.

MASLD disrupts lipid metabolism, leading to hepatic lipid accumulation and influencing various sources of reactive oxygen species (ROS) [42] so the influence of the antioxidant compounds for a closer physiological condition is more evident. MASLD courses also with inflammatory and abnormal lipid accumulation in hepatocytes. The pathogenesis situation for liver cells was also evaluated by using for the determination of major differences in fatty acid accumulation. Although previous cell protective effects were reported for PB2 incubation, this phenolic compound did not show significant differences in fat accumulation versus the OA induced control cells in ORO assay. However, in our case the results were significant for apple extract. Interestingly, this is not the first time that apple polyphenols are found to counteract lipid accumulation in fatty acid-induced intracellular lipid accumulation on HepG2 cells; in previous research these effects were linked via increased autophagy mediated by SIRT1 and activated LKB1/AMPK pathway [43]. These outstanding result matches with previous research in apple polyphenol extracts and their linkage with the alleviation of some related MASLD alterations in lipid metabolism [44, 45]. For a deeper understanding of the molecular influence in human steatosis cell model, certain RNA expression levels were measured by using qPCR.

PPARγ, CD36 and FAS genes are crucial for understanding the influence of polyphenols in cell lipid homeostasis modulation. PPARγ alterations have been linked to the pathogenesis of MASLD through insulin resistance, inflammation or even oxidative stress [11]. Moreover, some studies link high fat diets with an increase in expression of PPARγ and in consequence the close related free fatty acid receptor CD36 [46]. Activation of ERK-PPARγ signalling leads to increased hepatic fatty acid uptake and hepatocyte steatosis [47, 48]. The upregulation of these mechanisms in MASLD is important as a compensatory response of the body to the excessive fat accumulation in adipocytes during obesity. R1 Furthermore, FAS served as a key marker in hepatocytes with excessive fat accumulation because of its role in de novo lipogenesis [16].

PB2 may modulates the expression of PPARγ at higher doses but could not achieve significant differences for CD36 or FAS RNA expression levels. We should consider post-transcriptional changes to possibly explain some of those results such as with FAS, as well as alternative molecular pathways that influence lipid metabolism. However, this is an experimental limitation of this study, and more genes should be explored to complete these results.

Interestingly, our apple pulp extract achieves promising results and confirm the modulation in fat accumulation observed in ORO experiment. Despite more direct influence in oxidative stress related properties were linked to PB2, the complex mixture of many other polyphenols in the extract may explain this results. It is important to consider that certain combinations of phenolic compounds demonstrate synergistic effects, where their combined biological activity exceeds the sum of their individual effects [49].

The main limitation of this work is the lack of analysis of genes involved in lipid oxidation. Although the study provides relevant biochemical and functional data in the MASLD model, the absence of gene-expression profiling related to β-oxidation and mitochondrial lipid metabolism restricts a more comprehensive interpretation of how Amarilla de Ocubre apple pulp extract or PB2 modulate lipid processing in HepG2 cells. Key genes such as CPT1A and PPARα may be potential targets, given their reported modulation by polyphenols in similar hepatic steatosis models [50–52]. Including these genes would help clarify the specific molecular pathways underlying the observed effects.

Additionally, complementary experiments will be developed in future research to confirm the role of PB2 and the apple pulp extract, Amarilla de Octubre, in animal models and its impact in the homeostasis of fat metabolism. These are promising results for pharmaceutical and food industries considering the outstanding increment of worldwide population who develop MASLD caused by obesity.

## Supplementary Information

Below is the link to the electronic supplementary material.

Supplementary file 1 (DOCX 16.0 KB)

## Data Availability

No datasets were generated or analysed during the current study.
