# Supplementary material for: Procyanidin B2 and an autochthonous apple pulp extract modulate oxidative stress and PPARγ expression on an in vitro model of lipid steatosis in HepG2 cells
Source: J Physiol Biochem. 2026 Jan 23;82(1):4. doi: 10.1007/s13105-026-01151-9 (PMC12830479; doi:10.1007/s13105-026-01151-9)
Supplement: Supplementary file 1 — (DOCX 16.0 KB) [file 13105_2026_1151_MOESM1_ESM.docx]

**Supplementary information**

| **Gene ID** | **Sequence (5′ → 3′)** |
| --- | --- |
| **PPARy** | F AGA TGA CAG CGA CTT GGC AAT  R ACT CAG GGT GGT TCA GCT TC |
| **ACTB** | F CAT GTA CGT TGC TAT CCA GGC  R CTC CTT AAT GTC ACG CAC GAT |
| **FASn** | F AAG GAC CTG TCT AGG TTT GAT GC  R TGG CTT CAT AGG TGA CTT CCA |
| **CD36** | F GGC TGT GAC CGG AAC TGT G  R AGG TCT CCA ACT GGC ATT AGA A |

**Table S1.** List of primer sequences used for qPCR analysis in this study.
